# Effect of Variation in hemorheology between human and animal blood on the binding efficacy of vascular-targeted carriers

**DOI:** 10.1038/srep11631

**Published:** 2015-06-26

**Authors:** K. Namdee, M. Carrasco-Teja, M. B. Fish, P. Charoenphol, O. Eniola-Adefeso

**Affiliations:** 1 Department of Chemical Engineering, University of Michigan, Ann Arbor, MI 48109

## Abstract

Animal models are extensively used to evaluate the *in vivo* functionality of novel drug delivery systems (DDS). However, many variations likely exist *in vivo* between the animals and human physiological environment that significantly alter results obtained with animal models relative to human system. To date, it is not clear if the variation in hemorheology and hemodynamics between common animal and human models affect the functionality of DDS. This study investigates the role of hemorheology of humans and various animal models in dictating the binding efficiency of model vascular-targeted carriers (VTCs) to the wall in physiological blood flows. Specifically, the adhesion of sLe^A^-coated nano- and micro-spheres to inflamed endothelial cells monolayers were conducted *via* a parallel plate flow chamber assay with steady and disturbed red blood cells (RBCs)-in-buffer and whole blood flows of common animal models. Our results suggest that the ratio of carrier size to RBC size dictate particle binding in blood flow. Additionally, the presence of white blood cells affects the trend of particle adhesion depending on the animal species. Overall, this work sheds light on some deviation in VTC vascular wall interaction results obtained with *in vivo* animal experimentation from expected outcome and efficiency *in vivo in* human.

To date, vascular-targeted drug delivery remains of tremendous interest for use as a more effective treatment for many human diseases. To this end, several works have focused on characterizing the capacity for vascular-targeted drug carriers (VTCs) to markedly adhere to a targeted location either *via* various *in vitro* static and flow assays ranging in complexity from simple buffer to blood flows, e.g.^1^,^2^,^3^,^4^,^5^, or in various animal models of human diseases, e.g.^6^,^7^,^8^. *In vivo* assays are preferentially used due to the many challenges associated with recreating the complexity of the human *in vivo* physiological and biochemical environment with *in vitro* assays. Animals prominently used in drug delivery research include rodents, rabbits, pigs, dogs and monkeys^9^,^10^,^11^,^12^. While the pathology of many human diseases can be faithfully represented in these animals at the cellular, and in some cases organ levels, many of them have differences in their physiology relative to human (e.g. vessel size and hemodynamics) that may limit the extrapolation of the generated data to human physiology^13^, which may explain the repeated difficulty in translating a positive animal study into a successful human clinical trial.

The discrepancy in the physiology between laboratory animals and human is likely in the evaluation of particulate drug delivery systems (DDS) where the identified differences in hemorheology and hemodynamics, including differences in blood shear rates, RBC aggregation, and RBC geometry, may impact the distribution and performance of these systems/therapy. Indeed, several recent experimental and computational reports suggest that human RBCs have a significant influence on the binding efficiency of particles targeted to vascular wall under physiological blood flow *via in vitro* flow assays^5^,^14^,^15^,^16^. Specifically, small microparticles are shown to exhibit a high capacity to marginate (localize) and adhere to inflamed human ECs at the wall from human blood flow, while nanoparticles with sizes in the 100 to 500 nm diameter range exhibit limited margination. This observed size effect on the margination, and the subsequent adhesion, of particles is linked to the well-documented migration of RBCs away from the wall and alignment at the center of the flow, which creates a RBC-free layer, or cell free layer (CFL), near the wall. This central localization of RBCs causes microparticles (as well as micrometer-sized leukocytes and platelets) in blood flow to concentrate in the CFL *via* exclusion, enhancing their probability of collision and contact with the vascular wall^17^,^18^. However, a significant fraction of the nanoparticles in bulk blood flow are entrapped within the RBC core resulting in their low margination - i.e. they exhibit a low capacity to localize to the CFL^5^. The CFL width has been reported to vary from 2.5–7 μm in humans and some small animals depending on the hydrodynamic wall shear rate, vessel size, the volume fraction of RBC (percent blood hematocrit, % Hct), and the aggregability and deformability of the RBCs^19^,^20^,^21^,^22^. Therefore, any subtle differences in the physical properties of various animal RBCs may result in their differential lateral migration, and hence the formation of CFL, which would lead to variations in particle behavior and margination in different animal models.

Variations in RBC and blood characteristics between human and other common laboratory animals prominent in drug delivery research have been reported, including for mice, pigs, rabbits, dogs and monkeys. For example, the volume and aggregability of RBCs in mouse blood, a common animal species used for vascular-targeting research, are significantly smaller/lower than in humans^13^,^23^. Yet, to date, very limited works have investigated the impact of these variations in hemorheology between animal models and human on the hemodynamics of particulate carrier systems as it relates to their margination and adhesion to the vascular wall. Thus, this study aims to elucidate the role of RBC geometry in dictating the binding efficiency of spherical particles of various sizes in different blood flow patterns. Specifically, we evaluate the adhesion of inflammation-targeted polystyrene spheres in a parallel plate flow chamber (PPFC) to inflamed endothelial cells (ECs) from physiological flow of human, rabbit, pig and mouse RBCs-in-buffer (washed RBCs suspended in saline at fixed 40% Hct) and whole blood flow. The ligand density on particles and the magnitudes of blood flow were chosen such that particle adhesion exists in a transport-limited regime^2^; thereby allowing the assumption that particle adhesion directly correlates with particle margination. Overall, our results show that the capacity of VTCs to bind to the vascular wall is significantly influenced by blood flow types and most importantly, RBC size.

## Results

To study the effect of red blood cell (RBC) size (diameter and volume) on the localization and adhesion of VTCs to the vessel wall, sLe^A^-coated spherical particles, 0.2, 0.5, 2 and 5.7 μm diameter, were observed for their binding to inflamed HUVECs in laminar, pulsatile, and recirculating flow of human, pig, mouse and rabbit RBCs-in-buffer (RBC in buffer at 40% by volume or Hct) or whole blood in a PPFC. The HUVEC used were pooled from multiple donors such that the E-selectin expression, the major EC receptor for sLe^A^, is consistent from batch-to-batch as documented in a previous publication^24^. The same batch of cells are used for head-to-head comparison of particle adhesion as a function of particle and RBC sizes. The average diameter, volume, and the ratio of RBC volume to diameter (VDR) of the different animal RBCs used are summarized in Table 1.

### Effect of RBC size on particle adhesion in laminar flow

**Figure 1:**
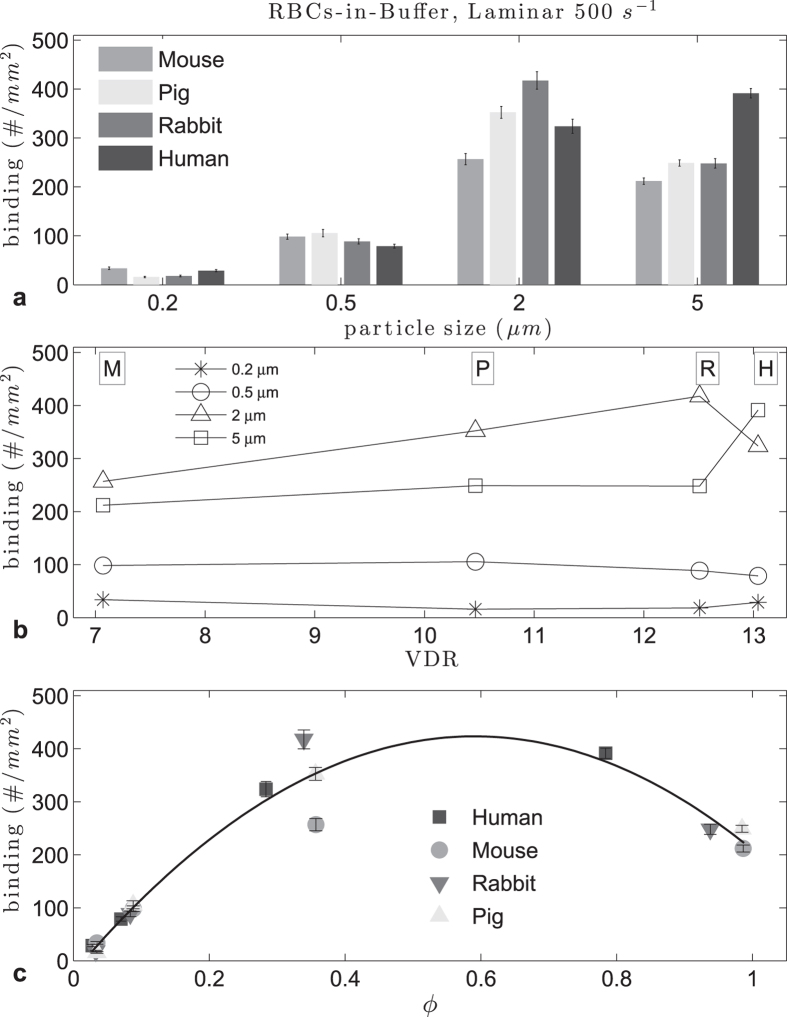
Adhesion of sLe^A^ -particles in human, pig, mouse and rabbit laminar RBCs-in-buffer flow, 40% Hct at 500 s^−1^ WSR: (**a**) histogram, (**b**) vs VDR and (**c**) ratio of particle diameter to RBC diameter ϕ (0.57 < ϕ_opt_ < 0.65, R^2^ = 0.94).

Figure 1 shows the average binding densities for sLe^A^ -particles observed in laminar RBCs-in-buffer flows at a wall shear rate (WSR) of 500 s^−1^. Overall, the adhesion density of microspheres at this WSR was significantly higher than the adhesion of nanospheres for all RBC types. For all but the 2 μm particles, adhesion in rabbit and pig RBC flows were not significantly different (see Supp. Table S1). When focused on the binding of spheres of a given size, the adhesion of the 2 μm spheres significantly increases as the RBCs-in-buffer changed from mouse to pig to rabbit following the order of increasing RBC volume (from 41 to 61 to 76 μm^3^, respectively). However, the adhesion of the 2 μm spheres in human RBCs-in-buffer flow was significantly lower than the density observed in both rabbit and pig RBC flows, despite the volume of the human RBC, 95 μm, being significantly larger than RBC volume in these animals. For the 5 μm spheres, the binding density increased significantly with the RBC change from mouse to pig but was not different between pig and rabbit RBCs-in-buffer flow. There was a significant increase in the 5 μm particle binding as the RBC changed from rabbit to human model. For the 500 nm spheres, particle binding density increased slightly with the RBC change from mouse to pig and then decreased with increase RBC volume as the RBC changed from pig, to rabbit to human. There was no significant difference in the adhesion of the 200 nm spheres with rabbit and pig RBCs (Supp. Table S1), while human and mouse RBCs yielded a slightly higher binding, but notably lower than all the other particle sizes. Overall, these observations suggest a trend in particle binding density with RBC volume, albeit a non-linear one.

To further probe how RBC geometry in the different animals affect the flow adhesion of particles of different sizes, we plotted the adhesion density data from Fig. 1(a) as a function of RBC diameter, RBC volume, and reduced volume (defined as the ratio of the averaged RBC volume, to the volume of a sphere with the same diameter as the RBC), and we found no appreciable trend for particle binding (Supplemental Figure S1). Conversely, as shown in Fig. 1(b), particle adhesion seems to have a linear relation to the RBC volume to diameter ratio (VDR), except again for larger particles with human RBCs. This, coupled with the trend observed for particle binding as a function of RBC volume for the different particle sizes (in Fig. 1(a)), would suggest that RBC volume is more important than RBC diameter in the dynamic interactions of RBC and particles in blood flow. However, the lack of a general trend in the effect of RBC volume on binding for all particles sizes prompted us to evaluate whether a correlation exists between particle and RBC size. Figure 1(c) shows the plot of particle binding density as a function of the ratio of particle diameter to RBC diameter, defined as ϕ, where we see a significant correlation. Specifically, we observe a quadratic relation in particle binding density when we normalized the particle sizes to the RBC size, similar to what was observed in^25^ for particle binding in human RBC flow as a function of particle size. Thus, we estimated an optimal ϕ range using a Monte Carlo method that incorporates the variability in particles sizes with the binding data using quadratic fits, as done in^25^. From the quadratic fit of the data in Fig. 1 at 40% Hct, with an averaged coefficient of determination R^2^ of 0.94, the optimal range for ϕ was 0.57 < ϕ < 0.65, which translates to an optimal particle diameter, d_opt_, range of 4.0 < d_opt_ < 4.6 μm for human RBCs, where d_opt_ is for the given flow regime and species. This agrees with the range of 3.8 < d_opt_ < 4.4 μm in 45% Hct given in^25^ for human RBCs in plasma, as the optimal size decreases with increased hematocrit. A head-to-head analysis of the adhesion of 4.08 μm diameter spheres in laminar human RBC-in-buffer flow, which is within the d_opt_ range for this flow type, shows that relative to the 2 and 5 μm spheres, the 4.08 μm spheres to have a higher adhesion (see Supplemental Figure S2). Conversely, the d_opt_ in this flow would be in the range of 3.3 < d_opt_ < 3.8 μm for mouse and pigs RBCs, and 3.5 < d_opt_ < 4.0 μm for rabbit RBCs. For completeness, we looked at the relation between particle binding and the ratio of particle volume to RBC volume, but the resulting data is not suited for curve fitting as the magnitude of these volume ratios were several orders apart and sparse. Overall, we find the quadratic correlation generated from the data in Fig. 1 can be generalized to any RBC geometry. Indeed, we see that the fit in Fig. 1 accurately predicts the binding densities for particles in cow RBCs-in-buffer flows (D = 5.5 μm)(see Supplemental Figure S3).

**Figure 2:**
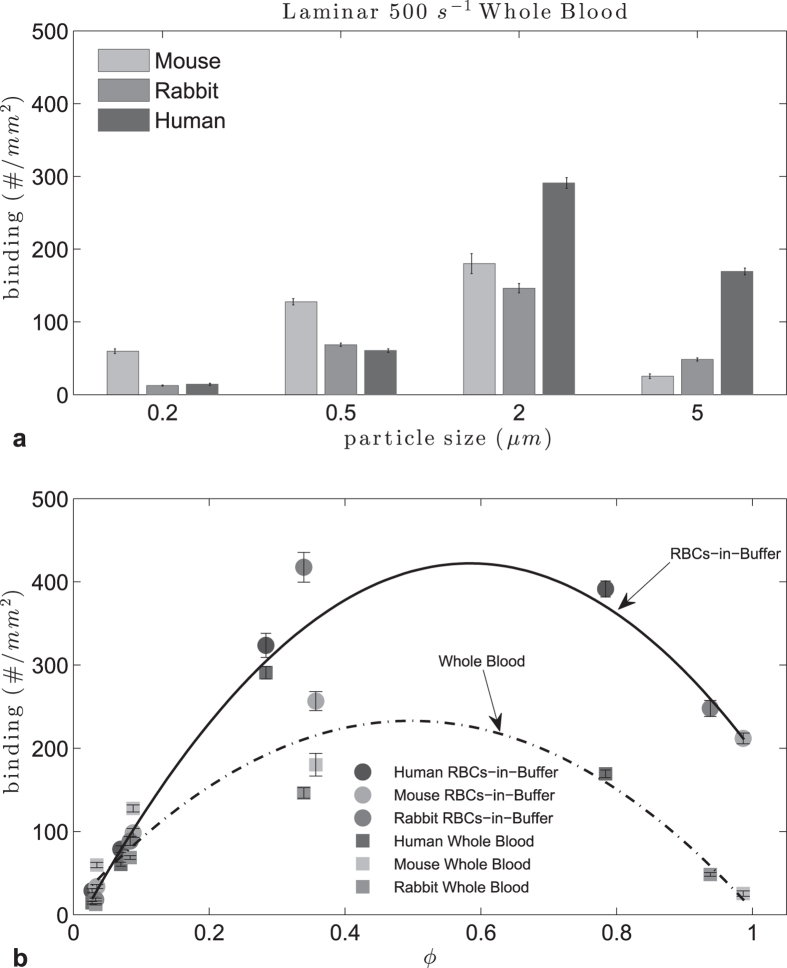
Adhesion of sLe^A^ -particles in human, mouse and rabbit laminar RBCs-in-buffer, 40% Hct, and whole blood flow at WSR 500 s^−1^ (**a**) histogram, (**b**) particle to RBC diameter ratio ϕ (RBCs-in-buffer 0.54 < ϕ_opt_ < 0.62, R^2^ = 0.93; Whole Blood 0.46 < ϕ_opt_ < 0.53, R^2^ = 0.75).

To see how the correlation between the particle and RBC size changes when all components of blood were present, we ran the experiments with mouse, rabbit, pig and human whole blood flow at 500 s^−1^ (Fig. 2). No binding occurred for particles of all sizes in pig whole blood which may be linked to interference of some components of pig blood plasma with particle binding^4^. Plasma protein composition, which may affect RBC aggregability, and plasma protein corona on particles, are known to vary between human and many animals^26^. There was a general decrease in microparticle adhesion in whole blood flow for the rest of the species relative to adhesion in RBCs-in-buffer flows, this being more pronounced for rabbit. Although a quadratic relationship was still observed between ϕ and the particle binding density, an R^2^ of 0.75 suggests that non-RBC components of blood, such as the number and size of white blood cells (WBCs), may play a significant role in particle binding. We have previously presented evidence that WBC collisions with bound microparticles, and not sLe^a^-mediated adhesion of particles to WBCs, resulted in the dislodging of the particles from the EC surface to ultimately result in their overall low adhesion level in human whole blood flow^25^. We confirmed this is also true for mouse and rabbit systems by comparing whole blood experiments with WBCs-removed blood experiments. A sample result is shown in Supplemental Figure S4 where the adhesion of 5 μm particles drastically increases in the WBC-removed blood relative to adhesion in whole blood flow under laminar flow. These results highlight the potential role of WBCs in particle adhesion to EC for microparticles in the blood flow of most common animal models and human whole blood flows. Nevertheless, the predicted d_opt_ for human RBCs of 3.36 < d_opt_ < 3.87 μm was again confirmed where 3.52 μm particles were shown to display significantly higher adhesion in laminar whole human blood flow than 2 and 5 μm spheres (Supp. Figure S2).

### Effect of RBC size on particle adhesion in pulsatile flow

**Figure 3:**
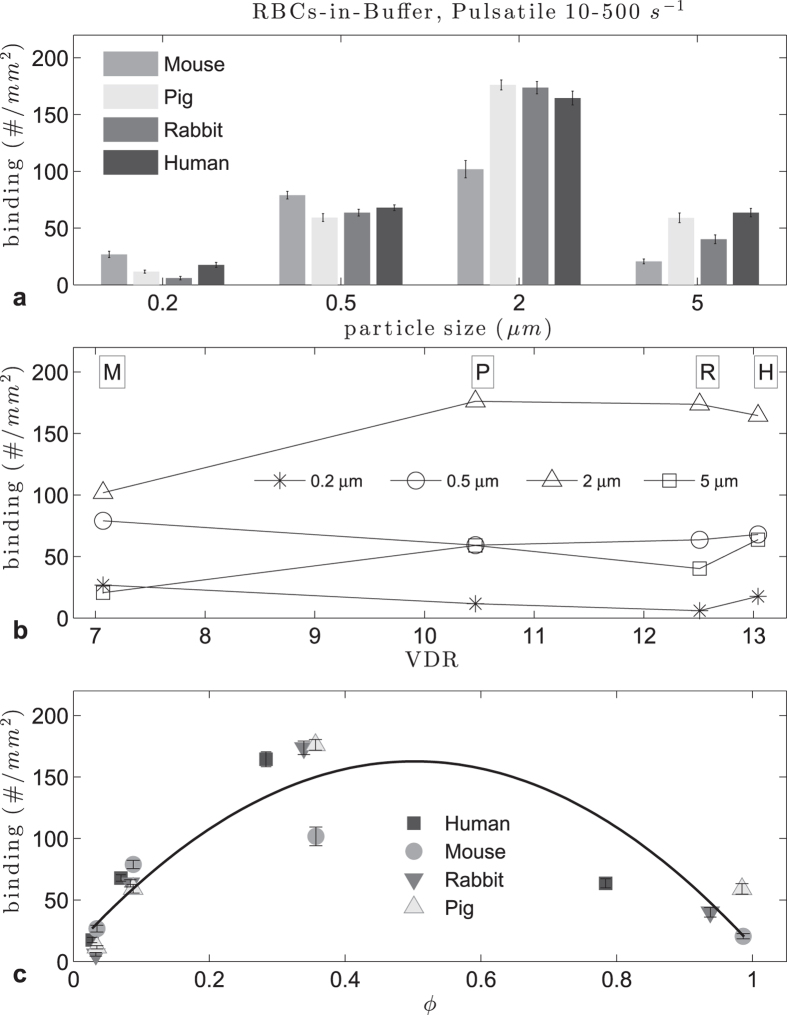
Adhesion of sLe^A^ -particles in human, mouse, pig and rabbit pulsatile RBCs-in-buffer flow at 40% Hct, 10–500 s^−1^ WSR (**a**) histogram, (**b**) plot vs VDR, (**c**) particle to RBC ratio ϕ (0.47 < ϕ_opt_ < 0.54, R^2^ = 0.78).

Particle binding trends were also observed in pulsatile flow. First we used a 10–500 s^−1^ WSR range (see Methods), with mouse, pig, rabbit and human RBCs. In general, the adhesion levels were lower for this flow profile than for laminar flow due to the smaller volume of blood, and hence a reduced number of particles that pass through the channel during the cycling between low and high shear rate for a fixed experimental time (Fig. 3(a)). For 0.5 and 2 μm particles, the difference between pig, rabbit and human RBCs-in-buffer flow adhesion levels was not significant (p > 0.01, see Supp. Table S1). All the animals followed a quadratic pattern with respect to the ratio ϕ, and no clear relation was found with RBC volume, RBC diameter, reduced volume or VDR, as with laminar flow. Overall, the adhesion of particles in pulsatile RBCs-in-buffer flow relative to particle size (Fig. 3(a)) followed the same trend as observed in laminar flow (Fig. 1(a)) with adhesion increasing as size increased from 0.2 to 2 μm. Interestingly, in pulsatile flow there was a stark reduction in particle adhesion when the size increased to 5 μm for all RBC types that was not observed in laminar flow. In the latter, the adhesion levels remained the same or slightly lower with the same size increase. It is possible that, on average, the CFL in pulsatile flow is smaller than in laminar flow due to cycling between periods of slow shear to high shear, which would create more RBC collisions with 5 μm spheres and impede their adhesion in pulsatile flow.

**Figure 4:**
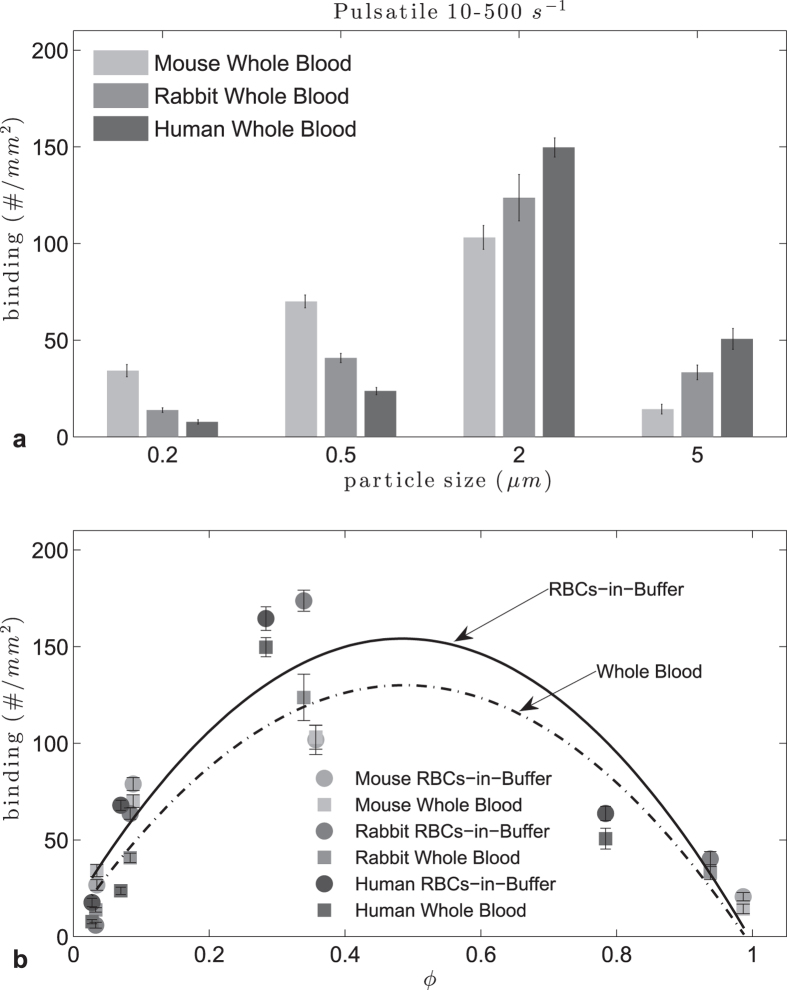
Adhesion of sLe^A^ -particles in human and mouse pulsatile RBCs-in-buffer and whole blood flow at 10–500 s^−1^ WSR (top) histogram, (bottom) particle to RBC ratio ϕ (RBCs-in-buffer 0.45 < ϕ_opt_ < 0.52, R_2_ = 0.78; Whole Blood 0.45 < ϕ_opt_ < 0.52, R^2^ = 0.81).

In whole blood experiments (Fig. 4), there was only a small decrease in adhesion compared to adhesion in RBCs-in-buffer flows, contrary to observation with laminar flow, likely due to the changes in shear force during flow that provides favorable conditions to overcome WBC interference with particle adhesion. The quadratic fit of adhesion density *vs* ϕ yielded an R^2^ = 0.78 for RBCs-in-buffer flow and R^2^ = 0.81 for whole blood flow. The optimum particle size was found to be in the range 3.4 < d_opt_ < 4.9 μm for humans, 2.8 < d_opt_ < 3.3 μm for rabbits and 2.7 < d_opt_ < 3.1 μm for mice and pigs in RBCs-in-buffer flow. In the case of whole blood the ranges are 3.2 < d_opt_ < 3.8 μm for humans, 2.6 < d_opt_ < 3.0 μm for rabbits and 2.8 < d_opt_ < 3.2 μm for mice. A change in the magnitude of the pulsatile flow to 120–1200 s^−1^ using human and mouse blood flow (Supp. Figure S5) yielded a similar adhesion pattern as in Fig. 4. The trend versus ϕ for this high WSR profile yielded again a quadratic fit with the same optimal ϕ range for both the whole blood and RBCs-in-buffer flow. Nevertheless, the optimal ranges for particle size was lower than that obtained for the lower WSR pulsatile flow, e.g. 3.4 < d_opt_ < 3.9 μm for humans and 2.7 < d_opt_ < 3.1 μm for mice.

### Effect of RBC size on particle adhesion in recirculating flow

**Figure 5:**
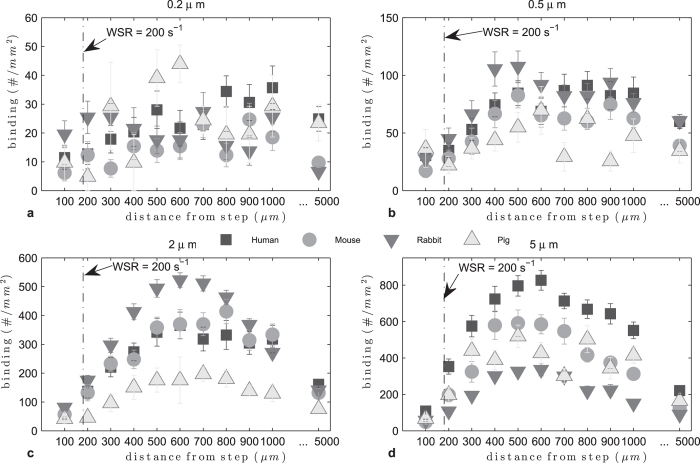
Adhesion of sLe^A^ -particles in human, mouse, rabbit and pig recirculating RBCs-in-buffer flow at 40% Hct, 200 s^−1^.

**Figure 6:**
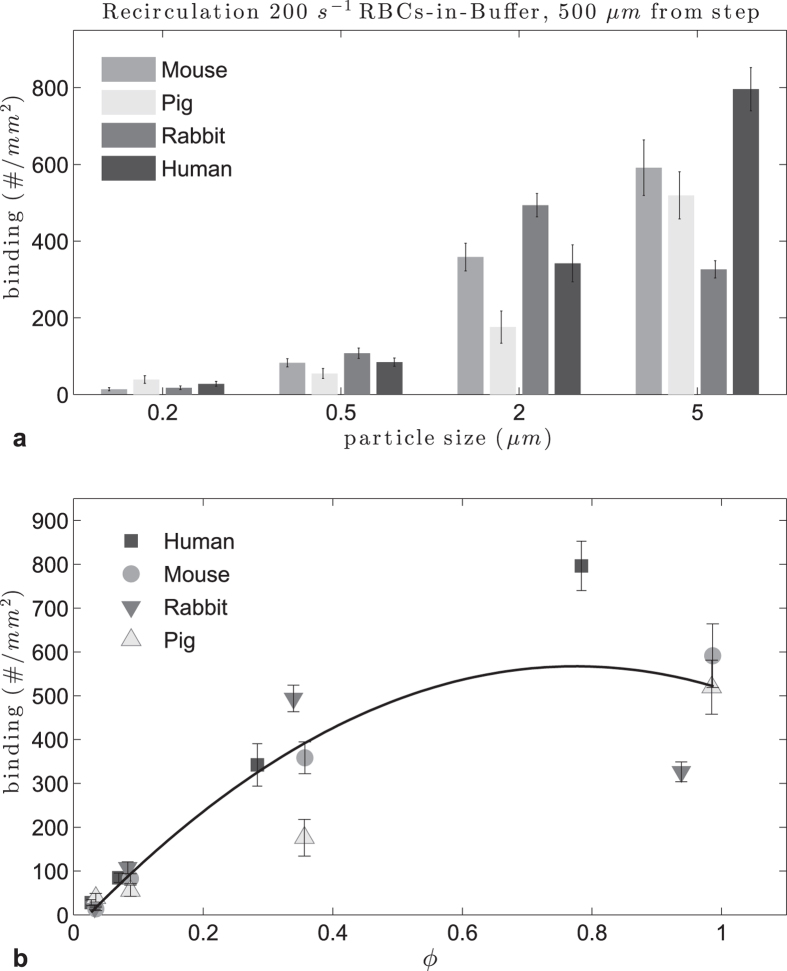
Adhesion of sLe^A^ -particles in human, mouse, rabbit and pig recirculating RBCs-in-buffer flow 40% Hct, 500 μm from step (**a**) histogram, (**b**) particle to RBC diameter ratio ϕ (0.74 < ϕ_opt_ < 0.85, R^2^ = 0.81).

Recirculating flow was achieved in the PPFC as described in the Methods section. In Fig. 5 adhesion densities in recirculating flow are plotted by particle size. The 0.2 and 0.5 μm particle adhesion levels were very low and no pattern can be discerned. For 2 μm particles, the adhesion levels increased compared to nanoparticles, with particle adhesion being highest in rabbit RBC flow than in the other species. For 5 μm particles, adhesion was the highest in human RBCs-in-buffer recirculating flow, as in laminar flow. In Supplemental Figure S6 we plot the adhesion levels by animal, where the difference between particle sizes is clearly appreciated. The adhesion peaked around 500 μm from the step for the microparticles; thus we evaluate the particle adhesion at this location as a function of particle size in the different RBC flows and evaluate whether a correlation exists for particle binding with the ratio of particle to RBC diameter (Fig. 6(a)). At this location, the particle adhesion density increased with particle size up to 5 μm for human, mouse and pig RBCs-in-buffer flow, while adhesion in rabbit RBC flow showed a decrease with the size increase from 2 to 5 μm. This observation is due to the 5 μm performing exceptionally well in this flow pattern for all RBC types relative to their performance in the other two flow profiles evaluated, which is in line with our previous report. This is likely due to the unique particle-RBC collision dynamics known to exist, and the resultant size dependent cellular/particle trapping within the vortex in recirculating flow^25^. Overall, there is still a quadratic pattern for particle adhesion with respect to ϕ though the fit was relatively poor with an R^2^ = 0.81.

**Figure 7:**
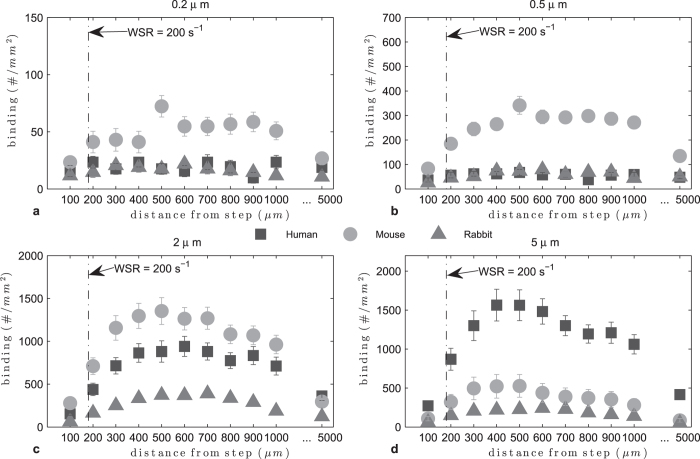
Adhesion of sLe^A^ -particles in human, mouse and rabbit recirculating whole blood flow at 40% Hct, 200 s^−1^.

**Figure 8:**
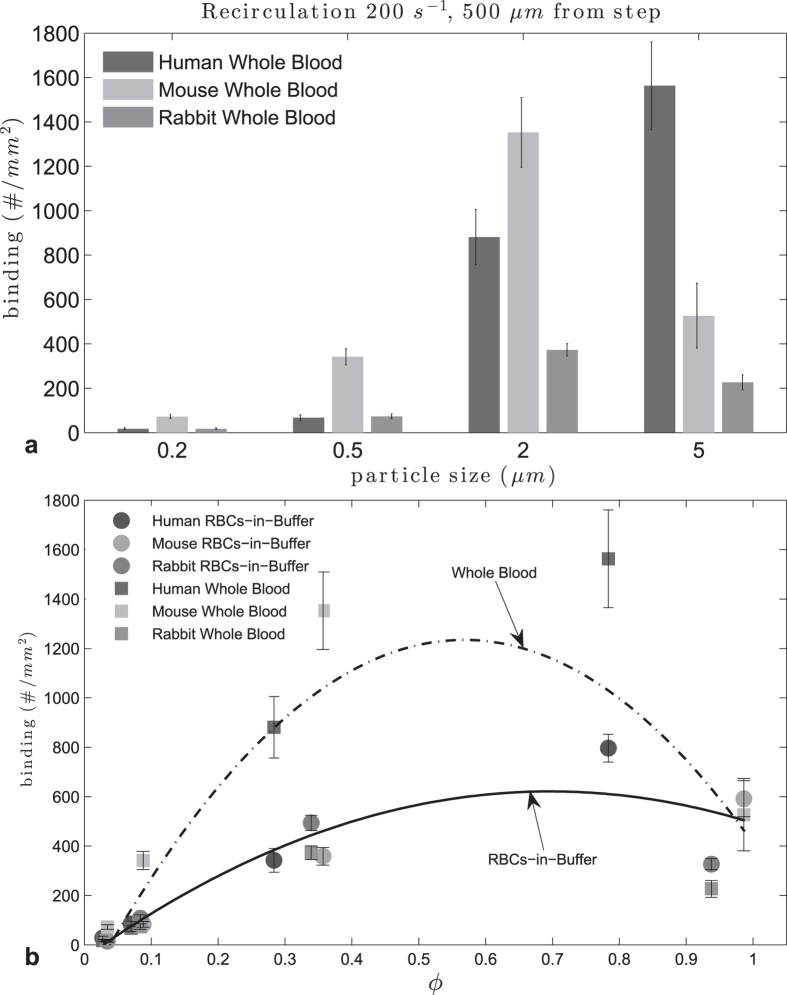
Adhesion of sLe^A^ -particles at 500 μm from step in human and mouse recirculating flow, 40% Hct, 200 s^−1^ : (**a**) histogram, (**b**) adhesion vs particle to RBC diameter ratio ϕ, (at 500 μm RBCs-in-buffer 0.65 < ϕ_opt_ < 0.74, R^2^ = 0.85, Whole Blood 0.53 < ϕ_opt_ < 0.61, R^2^ = 0.67).

For recirculating whole blood flow (Fig. 7), particle adhesion levels were higher relative to RBCs-in-buffer for all species. Also, both nanoparticle sizes exhibit enhanced adhesion in mouse whole blood relative to human and rabbit blood. The 2 μm spheres did particularly well in mouse and human blood whereas the 5 μm spheres exhibit exceptional binding in human blood compared to their adhesion in mouse and rabbit blood. Particle adhesion in rabbit whole blood was the lowest across the board, indicating a significant WBC effect, as also noted in the transition from RBCs-in-buffer to whole blood for rabbit in laminar flow (see Supp. Figure S7). Analysis of particle adhesion at 500 μm distance from the step shows a peak range for ϕ of 0.53 < ϕ < 0.61. This was smaller than the range estimated for flow of RBCs-in-buffer under the same flow condition (Fig. 8). This is likely due to the adhesion of the 5 μm spheres being significantly inhibited by the presence of WBCs in mouse and rabbit blood.

## Discussion

Due to the many advantages gained from utilizing targeted drug delivery in the treatment of diseases, several research works have been focused on the design and engineering of the optimal VTCs for localizing potent therapeutics to a target site in several human diseases, including cancer and cardiovascular diseases. To ensure the *in vivo* functionality of the designed VTCs, animal models of human diseases are utilized for testing despite the significant differences in blood rheology, hemodynamics and the vasculature structure of these animals compared to human^13^,^23^,^26^,^27^. Though correlation and the suggested allometric scaling factor of a particular animal to human exist for macroscopic scale differences (e.g. shear stress^26^,^27^), limited works have studied the potential implications of these variations in microscopic scale. This includes the effect of different size blood cells and cellular composition on the efficiency of VTCs relative to human physiology. The few works that exist relative to RBC size have primarily focused on platelet adhesion in high shear laminar flow^28^,^29^. Due to the significant influence of RBCs and, to a lesser extent, WBCs on particle margination in human blood flow as thoroughly described in^15^,^25^,^30^, it is likely that the distinct hemorheology that exists in animal models relative to humans can result in the differential pattern of localization and adhesion of VTCs of various sizes to the vascular wall. Herein, the adhesion of sLe^A^ coated spherical nano- and micro-particles to the inflamed HUVECs in flow of human, pig, mouse and rabbit RBCs-in-buffer (40% Hct RBCs suspended in saline) and whole blood was observed *in vitro via* PPFC assays with physiological shear conditions. Overall, the presented results showed that the binding efficiency of particles varies with the ratio of their dimension to the dimension of RBCs corresponding to different animal models, the flow pattern and blood constituents. The effect of different hematocrit was not investigated because it is not expected to affect the adhesion trend relative to particle size; we have previously shown that the trend does not change for different hematocrit in human^25^,^31^.

In buffer flow, the discrepancies in particle binding density are due to geometrical properties of the RBCs as there are no other blood complexities or variables present. We showed that the particle diameter to RBC diameter ratio, ϕ, gives us a good statistical estimate of the effect. Specifically, we found a quadratic relationship between ϕ and the adhesion density, which we anticipate is the product of two competing phenomena. On one hand, particle adhesion increases when particle size increases, which suggests an increase in margination with increase in particle size since adhesion assays were conducted in a transport-limited regime as described in a previous publication^2^. However, when the particle diameter is too large, the shear forces from the flow, as well as possible collisions with the RBC core flow, cause adhesion to decrease. This inflection point for particle adhesion relative to particle size changes with the RBC diameter. In order to confirm our findings, we tested the adhesion of particles with 4.08 and 3.52 μm average diameter, which lies in the optimal diameter (d_opt_) range for human RBCs-in-buffer and whole blood flow, respectively (see Supp. Table S2). The results clearly show a higher adhesion for the spheres in the predicted d_opt_ range compared to the 2 and 5 μm (Supp. Figure S2).

It is worth noting that since these adhesion experiments were observed in RBCs-in-buffer at fixed RBC volume fraction of 40% (which is consistent with the reported average hematocrit in human, mouse, rabbit and pig whole blood) at a fixed channel size, a larger number of RBCs with smaller volume, e.g. mouse RBCs, would be required to yield the same hematocrit as the larger RBCs in the same blood volume. Deformability is also known to strongly affect the alignment of RBCs under shear flow, i.e. less rigid RBCs tend to migrate to the center of flow while more rigid RBCs, less susceptible to the shear flow, are likely to stay adjacent to the wall^18^,^32^. At a fixed shear rate, deformability is affected by either the ratio between the RBCs diameter to height of the channel, sometimes refer red to as confinement^18^,^33^, or by the rigidity of the membrane of the RBC. As smaller RBCs will deform less than larger RBCs under the same flow conditions^34^, the CFL in the smaller RBC flows will probably be smaller^18^. Thus, the formation of RBCs at center of the flow is expected to be different for each species, and this affects the accuracy of ϕ_opt_. This accuracy could be improved if it were possible to remove experimental variables by adjusting the WSR with the membrane’s rigidity, and the channel height with the RBC diameter for each species, but that is experimentally challenging. For example, we observed differences in binding between mouse and pig RBCs-in-buffer flows for some experiments. This happens despite having a similar RBC average diameter and, thus, a similar ratio ϕ. This disparity is likely due to the two main differences between the two species RBCs: (1) mouse RBCs are more deformable than pig RBCs^34^ and (2) pig’s average RBCs volume is larger than mouse’s RBCs average volume (Table 1).

In whole blood flow, it is clear that the difference in adhesion levels depend strongly on the animal species, and therefore, on the specific blood constituents. These differences have a big impact in particle margination and, thus, it is expected that the quadratic fit of the ratio ϕ to particle adhesion density to be less accurate as it only takes into account geometric discrepancies. For instance, in the pig model, there was no binding in any of the flow profiles, which may be due to soluble factors in the pig’s plasma interfering with the targeting ligands’ ability to adhere to the wall^4^. For the other three species, human, mouse and rabbit, the particle to RBC diameter ratio ϕ, still yields a relative good fit, although the data points to the presence of WBCs extensively affecting particle binding in mouse and rabbit whole blood. We looked into the composition of the mouse and rabbit blood, specifically WBCs. The majority of WBCs in normal mouse and rabbit blood are lymphocytes, smaller than the RBCs, while normal human blood has more neutrophils, which are twice as large as human RBCs. This surprising large impact of the smaller-sized WBCs in the small animals on particle adhesion compared to the larger WBCs that dominate in human blood suggest that the size and concentration of WBCs in blood may be yet another factor that dictates particle adhesion and requires further studies to confirm.

Overall, our findings address important factors that help optimize the margination of micro- and nanoparticles in different species and highlight physical differences that may explain discrepancies between animal models and human clinical trials. Even though polystyrene particles are not biodegradable and, as such, cannot be used in human trials, our results showed the relevance of the relative size of the particle to the RBC, so that *in vivo* assays in animals have to be adjusted before applying them to humans. The optimal geometric conditions estimated can be transferred to biocompatible particles, such as PLGA. Finally, despite the fact that our results do not fully elucidate the role of plasma and other cells components, it clearly shows that these have an effect in the binding efficiency of VTCs, thus raising the awareness of potential deviation of animal results investigated *in vivo* and the expected outcome in human.

## Methods

### Preparation of Vascular-Targeted Spheres

Carboxylate-modified polystyrene spheres 0.20, 0.51, 2.07, 3.52, 4.08, and 5.72 μm in size (Bangs Laboratories Inc., Fishers, IN) were covalently coupled with NeutrAvidin protein (Pierce Biotech Inc., Rockford, IL) *via* carbodiimide (EDAC) chemistry as previously described^2^,^30^. Avidin-coated spheres were conjugated with biotinylated multivalent sialyl Lewis A (sLe^A^; GlycoTech, Gaithersburg, MD) in 50 mM PBS with 1% BSA and resuspended in blood at a fixed concentration of 5 × 10^5^ particles/mL, as previously described^2^. A fixed targeting ligand surface density of approximately 1000 sites/μm^2^ was achieved for all spheres by varying ligand concentration in the conjugation solution for each particle size. Sphere surface ligand densities were quantified *via* BD FACsCalibur^2^.

### Preparation of Human Endothelial Cell (EC) Substrate

Human umbilical vein endothelial cells (HUVECs) harvested from fresh umbilical cords were pooled and cultured in tissue culture flasks pretreated with gelatin (0.2% w/v)^35^. Umbilical cords were obtained from Mott Children’s Hospital (Ann Arbor, MI) under a Medical School Internal Review Board (IRB-MED) approved human tissue transfer protocol (HUM00026898). This protocol is exempt from informed consent per federal exemption category #4 of the 45 CFR 46.101.(b). For flow experiments, HUVECs were subcultured onto 30 mm glass cover slips pretreated with 1% w/v gelatin cross-linked with 0.5% glutaraldehyde until confluent^24^. Confluent HUVEC monolayers were activated with IL-1β at 1 ng/mL for 4 hours prior to use for experiments with sLe^A^ particles.

### Preparation of RBC-in-Buffer and Whole Blood (WB)

Human blood was collected from healthy adult donors *via* venipuncture into a syringe containing citrate anticoagulant (acid-citrate-dextrose, ACD), according to protocols approved by the University of Michigan Internal Review Board and in line with the standards set by the Helsinki Declaration. Appropriate written consent was obtained from all donors prior to blood draw. Human RBCs suspended in saline were prepared according to a previously published protocol^2^ where a 6% wt dextran-250 solution (1.4 mL/10 mL of blood) was added into anticoagulated whole blood to sediment RBCs from blood. RBCs collected from the bottom layer were washed with Dulbecco’s phosphate buffered saline (DPBS) and then resuspended in DPBS+ with 1% BSA (flow buffer) to achieve a 40% hematocrit (% Hct), i.e. volume fraction of RBCs. For animal RBCs-in-buffer, isolated pig, cow and rabbit RBCs were obtained commercially (Lampire Biological Lab, Pipersville, PA). For the murine RBC-in-buffer assays, mouse whole blood was collected *via* cardiac puncture with heparin as the anticoagulant from surplus mice generously provided by the breeding colony of Unit of Laboratory Animal Medicine (ULAM) according to a protocol approved by ULAM and University Committee on Use and Care of Animals (UCUCA) at the University of Michigan. The mouse blood was then centrifuged at 1000 g for 30 min to collect RBCs. Isolated RBCs were thoroughly washed in DPBS. The RBCs for all animals were then re-suspended in DPBS+ with 1% BSA at 40% Hct to yield RBCs-in-buffer. For whole blood experiments, animal whole blood containing heparin and human whole blood containing ACD were stored at 4 °C overnight before use.

### Flow Adhesion Experimental Set Up

A parallel plate flow chamber (PPFC) with a straight (for laminar and pulsatile flow) or a vertical step (for recirculating flow) gaskets forming the flow channel (GlycoTech, Gaithersburg, MD) were used for *in vitro* flow adhesion assays as described in a previous publication^2^. Briefly, a single straight gasket or a layered step channel was placed over an activated HUVEC monolayer cultured on a glass coverslip and vacuum-sealed to the flow deck to form the bottom adhesion substrate of the flow chamber assay. Vascular-targeted spheres of a given size suspended in RBCs-in-buffer or blood at a fixed concentration of 5 × 10^5^ spheres/mL were introduced into the flow channel from an inlet reservoir *via* a programmable syringe pump (KD Scientific, Holliston, MA).

For laminar flow assays, the wall shear rate (WSR), was computed using the approximation


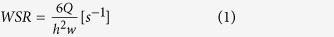


where Q (mL/min) is the volumetric flow rate through the channel, h is the channel height (254 μm, unless otherwise noted) and w is the channel width (1 cm unless otherwise noted).

For pulsatile flow, a programmable syringe pump was used to induce a pulsing profile in the horizontal PPFC as previously described^30^. Blood was pulsed forward with a fluctuating shear rate. Specifically, the syringe pump was set to run continuous loops with 4 s of low WSR to 2 s of high WSR for a total experimental time of 5 minutes. The flow rate Q was set to a minimum of 0.0645 mL/min (WSR = 10 s^−1^) to a maximum of 3.225 mL/min (500 s^−1^) for the first set of experiments; and a minimum of .774 mL/min (WSR = 120 s^−1^), and maximum of 7.74 mL/min (1200 s^−1^) for the second set of experiments. The WSR(t) was computed from Q(t) using equation (1).

Recirculating flow was generated *via* a vertical-step gasket (VSFC)^36^ that formed entrance and main channel heights of 127 μm and 508 μm (0.5 cm width), respectively, as previously described^30^. The sudden expansion at the step generates recirculation in flow where a two-dimensional flow with a parallel (V_x_) and a normal flow velocity (V_z_) with respect to the channel bottom wall is established. The recirculation vortex extends from the step to a reattachment point where V_x_ = 0 and only a negative V_z_ (pointing toward the bottom wall) is present. Beyond the reattachment, flow moves forward, and a laminar profile is reestablished at far downstream. The flow rate through the VSFC was set such that a laminar WSR of 200 s^−1^ was achieved in the main chamber, and the total experimental time was set again to 5 minutes.

Flow adhesion assays were observed on a Nikon TE 2000-S inverted microscope fitted with a digital camera (Photometrics CoolSNAP EZ with a Sony CCD sensor). Digital recording of experiments was *via* Metamorph analysis software. All adhesion experiments were conducted at 37 °C and each cell monolayer was used once.

### RBC Volume Measurement

To determine the average RBC volume for all animal and human blood, 15 mL of whole blood was spun down at high speed, 1000 rpm, for 30 min, then the WBC layer and plasma were removed. 1 mL of RBCs, from the bottom part of RBC layer (assuming RBCs were completely packed), were collected and diluted in DPBS buffer (2000X, 5000X and 10,000X). RBCs concentrations were counted by hemocytometer. RBC volumes are calculated based on RBCs/volume. All processes were repeated 3 times.

### Data analysis

Particle binding density (#/mm^2^) in laminar and pulsatile flow is obtained by manual count of the number of particles bound on the cell monolayer after 5 minutes of flow and dividing this number by the area of the field of view (20x magnification, Area = 0.152 mm^2^, unless otherwise stated). For recirculating flow experiments, the number of particles bound downstream of the step channel was counted in 100 μm intervals (area of strip = 0.034 mm^2^), as previously described^30^. Each data point represents an average of at least three experiments and includes at least five fields of view per experiment. Standard error bars were plotted. Differences in adhesion levels were analyzed using a student t-test and one-way ANOVA with Tukey post-test. A value of p < 0.01 was considered statistically significant.

To estimate a range of optimal particle size for each flow condition, we used a Monte Carlo method that incorporated the variability in particle size with the binding data as described in^25^.

## Additional Information

**How to cite this article**: Namdee, K. *et al.* Effect of Variation in hemorheology between human and animal blood on the binding efficacy of vascular-targeted carriers. *Sci. Rep.* **5**, 11631; doi: 10.1038/srep11631 (2015).

## Supplementary Material

Supplementary Information

## Figures and Tables

**Table 1:** Red blood cell average size of human and different animal species 37.

Species	Diameter (μm)	Volume (μm^3^)	VDR
Human	7.3	95	13.88
Rabbit	6.1	76	13.35
Pig	5.81	61	11.17
Mouse	5.8	41	7.55
