# Supplementary material for: Effect of Variation in hemorheology between human and animal blood on the binding efficacy of vascular-targeted carriers
Source: Sci Rep. 2015 Jun 26;5:11631. doi: 10.1038/srep11631 (PMC4481524; doi:10.1038/srep11631)
Supplement: Supplementary Information [file srep11631-s1.pdf]

# Effect of variation in hemorheology between human and animal blood on the binding efficacy of vascular-targeted carriers: **Supplementary Information**

*K. Namdee, M. Carrasco-Teja, M. B. Fish, P. Charoenphol and O. Eniola-Adefeso*

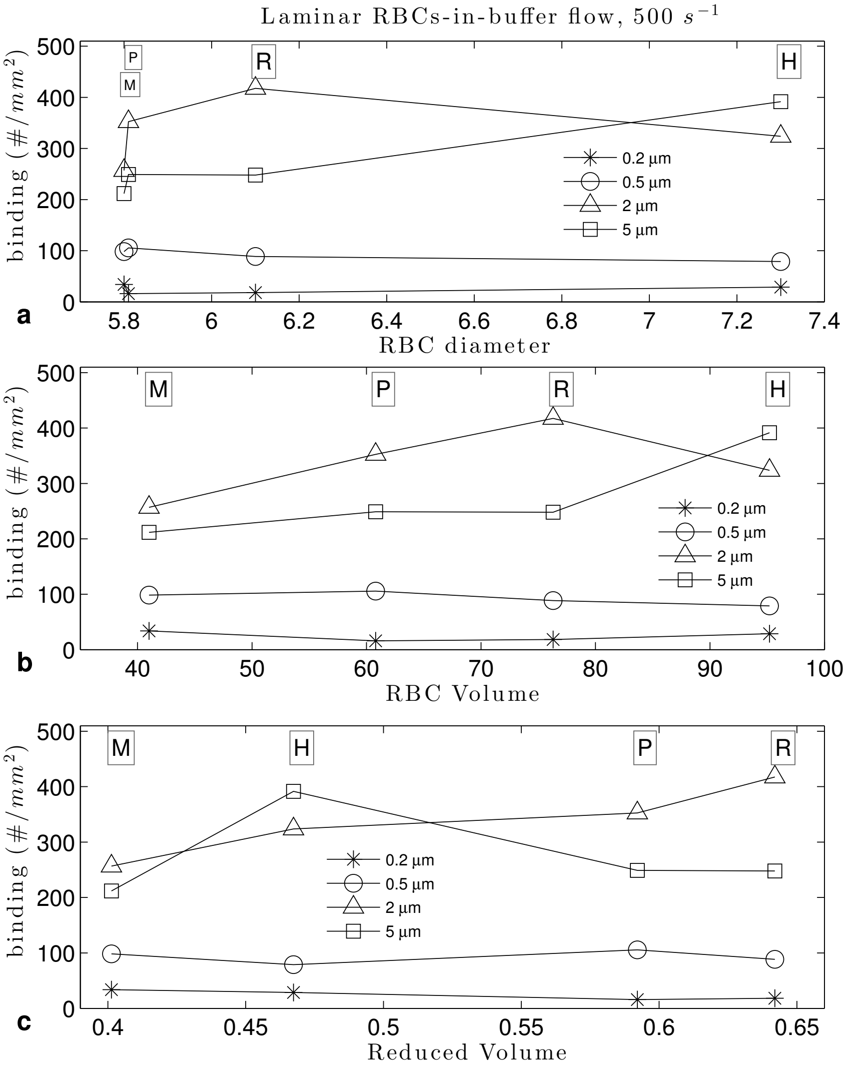


Figure S.1: Adhesion of sLe$^A$-particles in human, mouse, rabbit and pig laminar RBCs-in-buffer flow at $500s^{-1}$ WSR (a) vs RBC diameter ($d_{RBC}$), (b) vs RBC volume ($V_{RBC}$) and (c) vs reduced volume $\left(\frac{V_{RBC}}{\frac{4\pi}{3}\left(\frac{d_{RBC}}{2}\right)^3}\right)$.

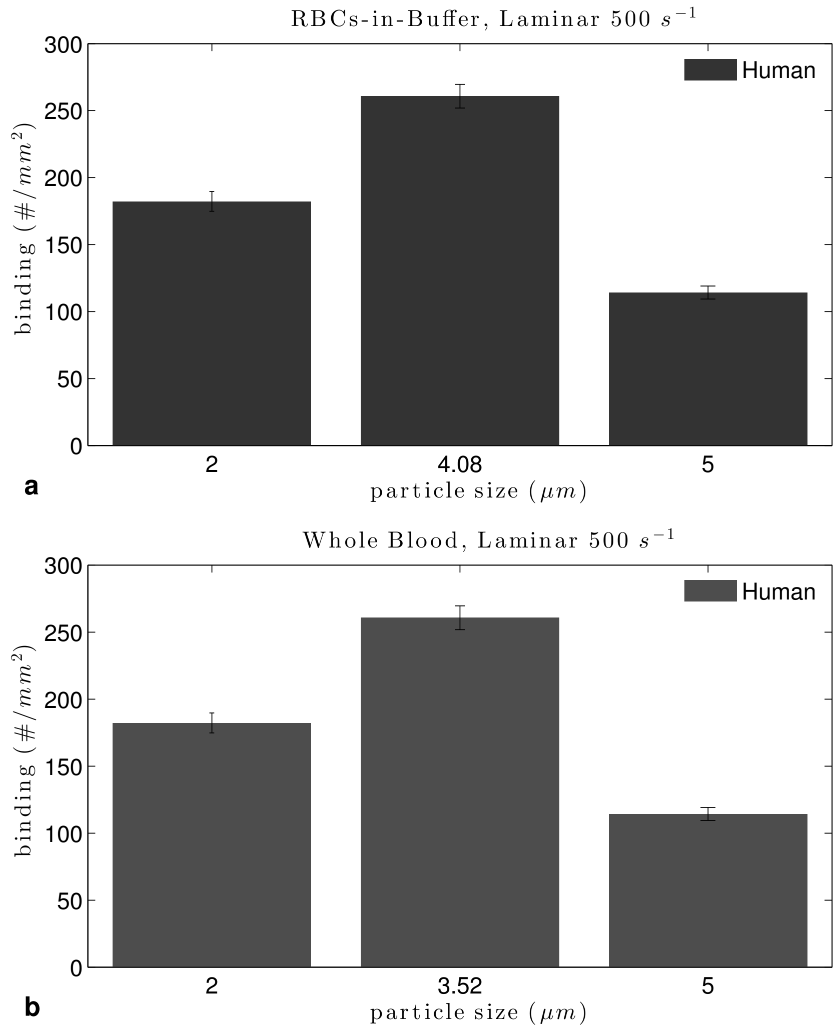


Figure S.2: Adhesion of sLe$^A$-particles in human laminar flow at 40% Hct, 500 $s^{-1}$ in the $d_{opt}$ range (a) RBCs-in-buffer (b) whole blood.

Table S.1: $p$-value from balanced one-way ANOVA, only $p > 0.01$ are shown.

| Figure | Significance (ANOVA) |
|---|---|
| 1 | rabbit vs mouse $2\mu m$ $p = .05$;<br>human vs rabbit $0.2\mu m$ $p = 0.3$, $0.5\mu m$ $p = 0.018$ |
| 2 | rabbit vs pig $0.2\mu m$ $p = 0.38$, $0.5\mu m$ $p = 0.14$, $5\mu m$ $p = 0.94$;<br>rabbit vs human $0.5\mu m$ $p = 0.21$ |
| 3 | rabbit, pig vs human $5\mu m$ $p = 0.0152\mu m$ $p = 0.30$, $0.5\mu m$ $p = 0.11$;<br>human vs pig: $0.2\mu m$ $p = 0.033$;<br>human vs rabbit $0.2\mu m$ $p = 0.012$ |
| 4 | rabbit vs human $2\mu m$ $p = 0.3134$, $5\mu m$ $p = 0.16$ |
| S.5 | human RBCs-in-buffer vs human Whole Blood $0.2\mu m$ $p = 0.48$, $2\mu m$ $p = 0.067$;<br>mouse RBCs-in-buffer vs mouse Whole Blood $5\mu m$ $p = 0.76$ |
| 6 | human vs rabbit vs mouse $0.2\mu m$ $p = 0.084$, $0.5\mu m$ $p = 0.14$ |

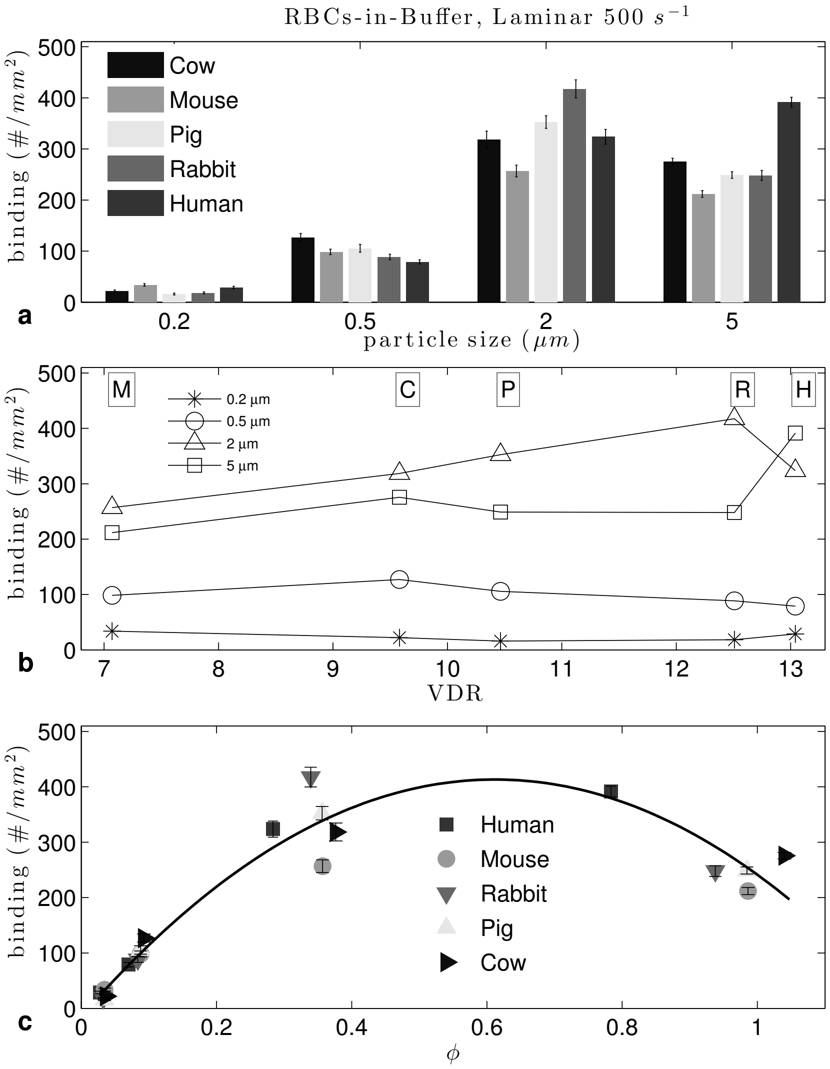


Figure S.3: Adhesion of sLe$^A$-particles in human, mouse, rabbit, pig and cow laminar RBCs-in-buffer flow at $500s^{-1}$ WSR (a) histogram, (b) vs VDR and (c) ratio of particle diameter to RBC diameter, $0.57 < \phi_{opt} < 0.65$ ($\bar{R}^2 = 0.92$).

Table S.2: Optimal particle diameter ($d_{opt}$) in different flows for each animal species.

| RBCs-in-Buffer | Human | Mouse | Rabbit | Pig |
|---|---|---|---|---|
| Laminar $500s^{-1}$ | 4.16–4.74 $\mu m$ | 3.31–3.77 $\mu m$ | 3.47–3.96 $\mu m$ | 3.31–3.77 $\mu m$ |
| Pulsatile 10-$500s^{-1}$ | 3.43–3.94 $\mu m$ | 2.73–3.13 $\mu m$ | 2.87–3.29 $\mu m$ | 2.73–3.13 $\mu m$ |
| Recirculating $500s^{-1}$ | 5.40–6.21 $\mu m$ | 4.29–4.93 $\mu m$ | 4.51–5.19 $\mu m$ | 4.30–4.94 $\mu m$ |

| Whole Blood | Human | Mouse | Rabbit |
|---|---|---|---|
| Laminar $500s^{-1}$ | 3.36–3.87 $\mu m$ | 2.67–3.07 $\mu m$ | 2.81–3.23 $\mu m$ |
| Pulsatile 10-$500s^{-1}$ | 3.29–3.80 $\mu m$ | 2.61–3.02 $\mu m$ | 2.75–3.17 $\mu m$ |
| Recirculating $500s^{-1}$ | 3.87–4.45 $\mu m$ | 3.07–3.54 $\mu m$ | 3.23–3.72 $\mu m$ |

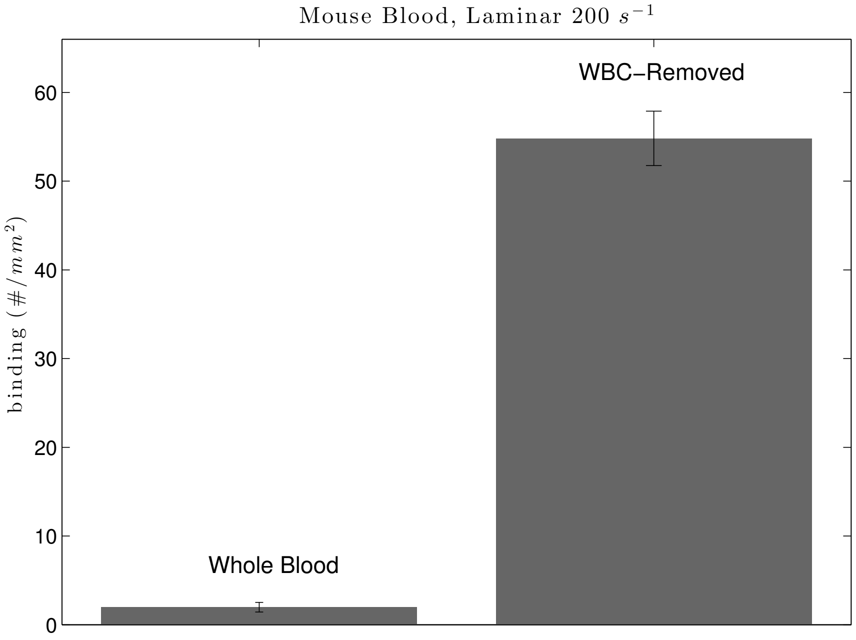


Figure S.4: Adhesion of $2\mu m$, sLe$^A$-PLGA-particles in laminar flow $200s^{-1}$ WSR, mouse blood.

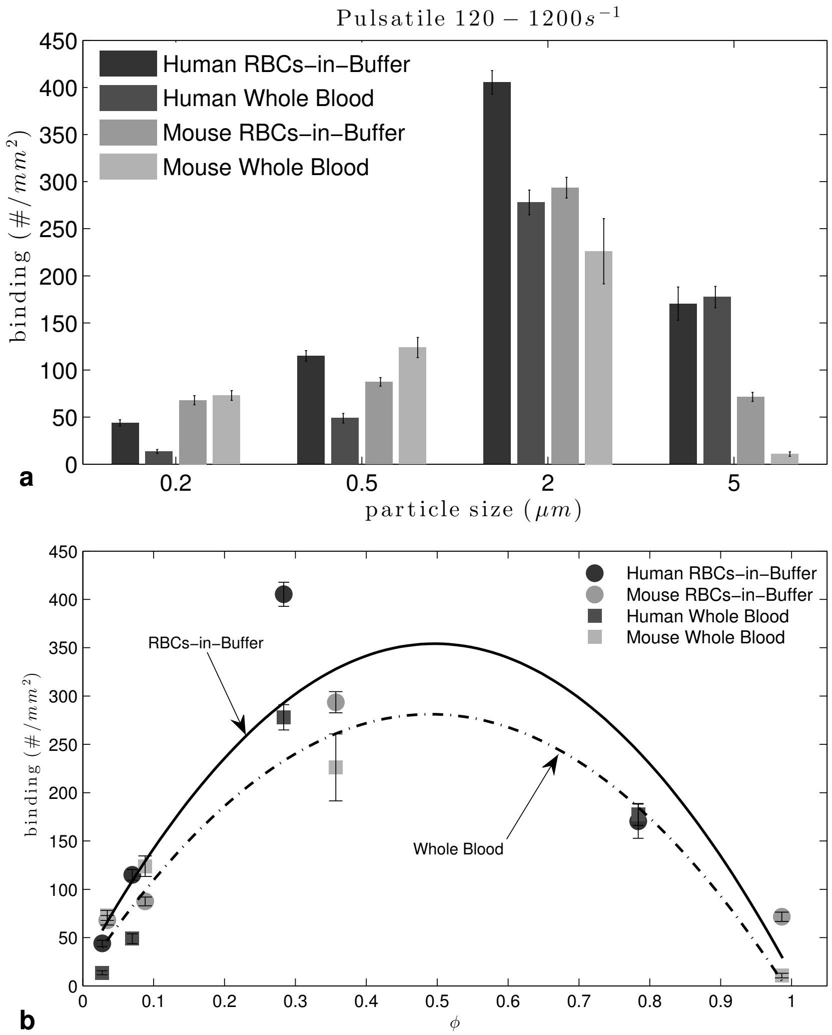


Figure S.5: Adhesion of sLe$^A$-particles in human and mouse in pulsatile RBCs-in-buffer and whole blood flow at 40% Hct,120-1200 $s^{-1}$ WSR: (top) histogram, (bottom) particle to RBC ratio (RBCs-in-buffer $0.46 < \phi_{opt} < 0.53$, $\bar{R}^2 = 0.80$; Whole Blood $0.46 < \phi_{opt} < 0.53$, $\bar{R}^2 = 0.91$).

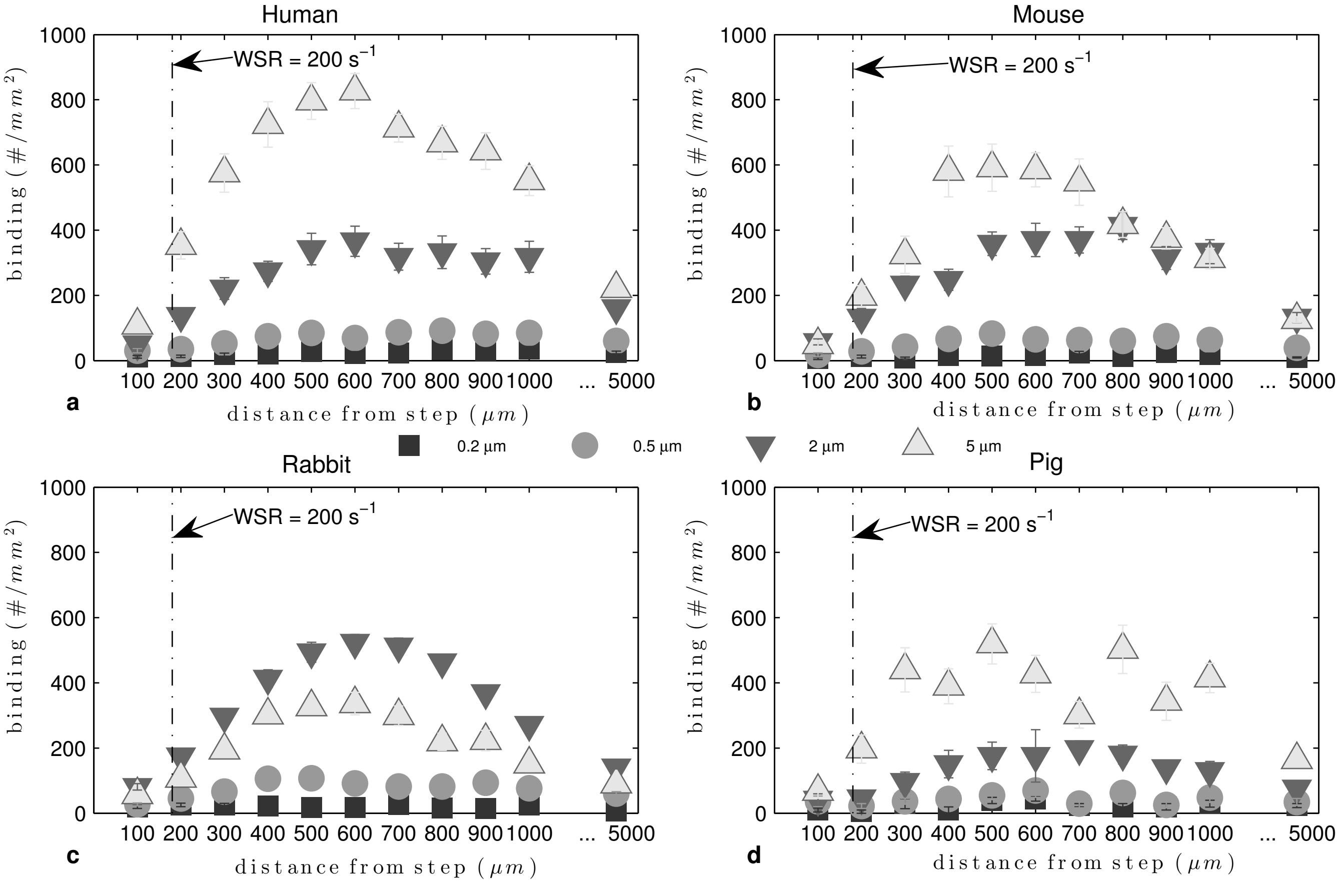


Figure S.6: Adhesion of sLe$^A$-particles in human, mouse, rabbit and pig in recirculating RBCs-in-buffer flow at 200 $s^{-1}$.

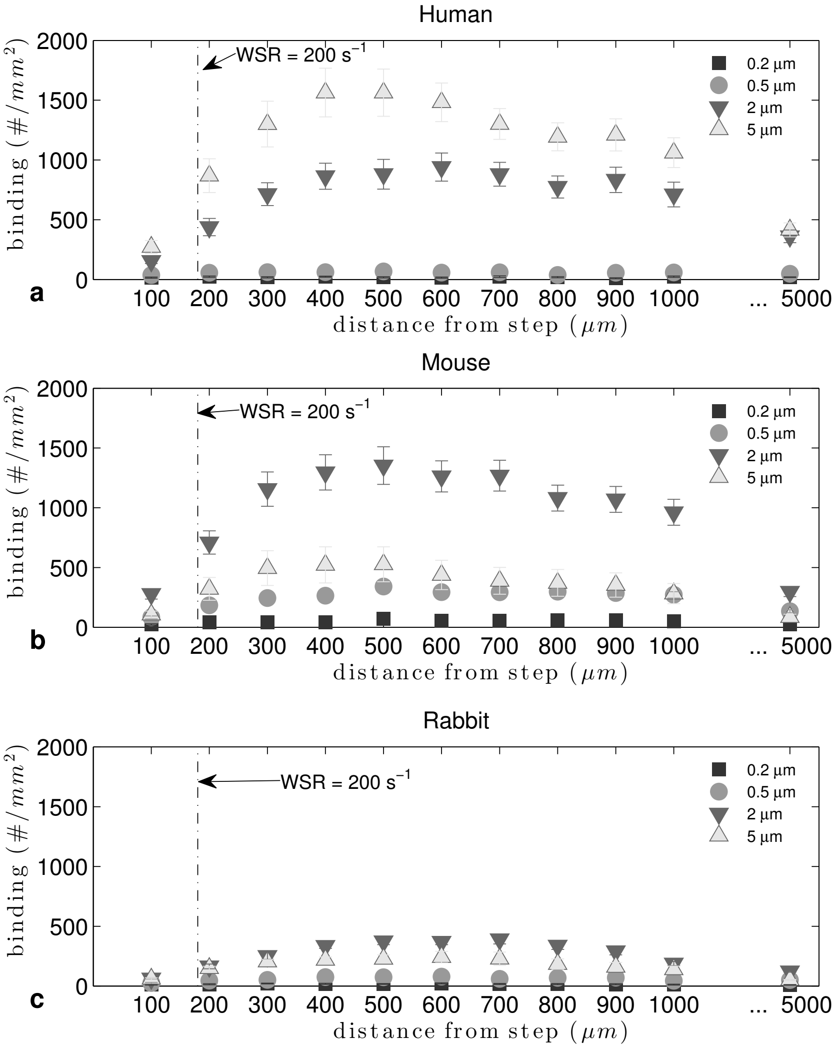


Figure S.7: Adhesion of sLe$^A$-particles in human, mouse and rabbit recirculating whole blood flow at 40% Hct, 200 $s^{-1}$ grouped by species.
